# Risks of Occupational-Related Adverse Events (ORAEs) and Effect of Bundled Interventions Among Health Care Workers in Novel Coronavirus Pneumonia Wards

**DOI:** 10.1017/dmp.2021.237

**Published:** 2021-07-23

**Authors:** Tinggang Luo, Yao Guo, Ying Shi, Yujian Song, Wenchao Xu, Jianping You

**Affiliations:** 1 Department of Infection Control, Characteristic Medical Center of the Chinese People’s Armed Police Force, China; 2 The Graduate School of the Chinese PLA General Hospital, Beijing, China; 3 Institute of Neurotrauma and Repair, Characteristic Medical Center of the Chinese People’s Armed Police Force, China; 4 Institute for the Prevention and Treatment of Skin Diseases, Characteristic Medical Center of the Chinese People’s Armed Police Force, China; 5 Department of Infectious Diseases, Southwest Hospital, Army Medical University (The Third Military Medical University), China

**Keywords:** adverse events, COVID-19, health care workers, occupational exposure, personal protective equipment

## Abstract

**Objective:**

The aims of the study were to investigate the burden for health care workers (HCWs) who suffer from occupational-related adverse events (ORAEs) while working in contaminated areas in a specialized hospital for novel coronavirus pneumonia, to explore related risk factors, to evaluate the effectiveness of bundled interventions, as well as to provide scientific evidence regarding the reduction of risks concerning ORAEs and occupational exposure events.

**Methods:**

The study was completed using a special team of 138 HCWs assembled for a specialized hospital for novel coronavirus pneumonia in Wuhan, dated from February 16 to March 26, 2020. The incidence of occupational exposure was determined by data reported from the hospital, while the prevalence of ORAEs was derived from questionnaire results. The relation coefficients of ORAEs and the variable potential risk factors are analyzed by logistic regression. After the risk factors were identified, targeted organized intervention was implemented and chi-square tests were performed to compare the incidence of occupational exposure and the prevalence of ORAEs in contaminated areas before and after the interventions.

**Results:**

Ninety one out of 138 (65.94%) had reported ORAEs with 300 (27.96%) cases of ORAEs being recorded in a total of 1073 entries into contaminated areas. The prevalence of different ORAEs include 205 tenderness (24.73%), 182 headache/dizziness (21.95%), 138 dyspnea (16.65%), 130 blurred vision (15.68%), and 95 nausea/vomiting (11.46%). Personal protective equipment (PPE) is significantly associated with ORAEs in contaminated areas (*P* < 0.05). Among non-PPE-related factors, insomnia is associated with the majority of ORAEs in contaminated areas. Significant differences were achieved after organized interventions in the incidence of occupational exposure of HCWs (χ^2^ = 39.07, *P* < 0.001) and the prevalence of ORAEs in contaminated areas (χ^2^ = 22.95, *P* < 0.001).

**Conclusion:**

During the epidemic period of novel severe respiratory infectious disease, the burden of the ORAEs in contaminated areas and the risk of occupational exposure of HCWs were relatively high. In time, comprehensive and multi-level bundled interventions may help decrease the risk of both ORAEs and occupational exposure.

## Introduction

Occupational exposure carries significant risk of threatening the safety of health care workers (HCWs). During the severe acute respiratory syndrome (SARS) outbreak in 2003, 931 HCWs were infected, accounting for 19% of all clinically diagnosed cases^1^; whereas, during the novel coronavirus disease (COVID-19) pandemic, over 1700 HCWs in Wuhan have been infected and developed COVID-19-related diseases due to various types of occupational exposures.^2^ Protecting the safety and well-being of frontline HCWs has drawn growing concerns all over the world. In clinical practice, the occupational exposure events during the pandemic include not only respiratory exposure, but also blood-related and other mucosal exposures. In the hospitals specializing exclusively on COVID-19 medical care in the epidemic regions, occupational exposure has become one of the most critical risks threatening the lives and health of HCWs.^3,4^ Based on the information of an unpublished report on occupational exposure from a COVID-19 hospital in Wuhan, the frequency of occupational-related adverse events (ORAEs) in contaminated areas appeared relatively high. Large scale studies on ORAEs in contaminated areas and the risk of occupational exposure for HCWs were rare before the COVID-19 pandemic. This study attempted to identify the prevalence of ORAEs in HCWs working in the contaminated areas, to explore the incidence and risk factors of various adverse events (AEs), and to determine the effectiveness of bundled interventions during the outbreak of emerging severe respiratory infectious diseases, providing evidences to establish reasonable and scientific interventions to decrease the risk of ORAEs.

## Methods

### Study Subjects

A total of 143 HCWs in a special team urgently assembled for a COVID-19 specialized hospital to work in Wuhan from February 16 to March 26, 2020, were asked to join this investigation by completing a questionnaire. The inclusion criteria are having a clear awareness, the ability to understand the content of the questionnaire, and voluntarily signing an informed consent. Incomplete and obviously inappropriate questionnaires were excluded. A total of 138 (96.5%) valid questionnaires were returned.

The information obtained from the questionnaire included age, gender, specialty and qualification of the HCW, weather temperature (highest and lowest) of the workday, number of times entering as well as the duration in the contaminated area, ORAEs. Self-identified causes of ORAEs were documented by the study participants, including personal protective equipment (PPE)-related factors (eg, mask suffocation, goggle oppression, goggles/eyeglasses fogging, and protective clothing sultriness) and non-PPE-related factors (eg, insomnia, car sickness, dysphoria, dyspepsia, and overfatigue).

The clinical presentations included in the ORAEs are nausea, vomiting, dizziness, headache, tenderness, palpitation, dyspnea, blurred vision, falls, epistaxis, and stick/cut injuries. Tenderness was defined as pain of skin caused by redness, blisters, or even ulcer at the site of facial bony eminence (such as nose, zygoma, forehead, and back of the auricle), due to wearing the mask, goggles, and protective face screen. Occupational exposure events were defined as needle stick/cut exposure, broken skin/mucosal exposure, and respiratory exposure during occupational activities.

### Methods

This is a self-controlled study. The participants were given bundled interventions. The differences in the incidence of occupational exposure events and prevalence of ORAEs before (February 16 to February 22, 2020) and after the interventions (February 23 to March 26, 2020) were compared.

### The Bundled Interventions

Based on the factors discovered from our previous experience in the infectious disease unit and at the early stage of the special team, each participant received bundled interventions (Tables 1 and 2).

**Table 1.** Protocols used for the bundled interventions

Subjects	Main Contents
Professional consultation	Problem-based, proactive instructions and discussions offered by the infection control experts in the special team.
Training	Focus on isolation behaviors (eg, hand hygiene, sterilization, respiratory hygiene, and cough-manners) and the appropriate utilization of PPE with repeated and enhanced training by various approaches such as posters, standard operating procedures, electronic documents/web pages, warning signs, problem-oriented discussions, and seminars.
Signs and posts	Warning signs distinguished by colors.
Video surveillance	High risk locations monitored by designated infection control personnel so that emergency conditions can be timely handled.
Entrance management	Help to recognize AE-related factors* by filling forms before entering contaminated areas. HCWs should take shower and change gowns before entering the isolated area.
Pocketbook	Providing all personnel with pocketbooks that contain knowledge for self-protection, hospital partition, and emergency responses.
Drills	Simulating occupational exposure events, medical wards have drills based on established protocols that are video-recorded for further study.
Supervision	Cross supervision performed by infection control personnel on a regular basis.
Comments	Highlighting common and prominent problems, promoting advanced prevention and control techniques and experiences.
Assessments	All HCWs should complete repeated assessments.
Psychological support	Psychological counseling.

*Details in Table 2.

PPE: personal protective equipment; AE: adverse event; HCWs: health care workers.

**Table 2.** Checklist for prevention of ORAEs, to be completed before entering the contaminated areas

	Confirm	Failed
Rested for more than 1 hour after taking shuttle bus	□	□
Sleeping time more than 8 hours today	□	□
No abdominal fullness and nausea	□	□
Anti-fog goggles/anti-fog measures	□	□
No headache	□	□
Working duration < 4 hours in a contaminated area	□	□
Expect guided wearing of PPE on the spot	□	□

### Data Processing and Statistical Analysis

All investigations were implemented via Wenjuanxing System, a popular and largest online questionnaire platform in China. Statistical analyses were performed with SPSS statistical software version 25.0 (IBM Corp, Armonk, NY). Quantitative data were shown with $\overline x \pm s$ according to distribution characteristics. Count data were represented by n (%). Two independent samples, t and chi-square tests, were used in group comparisons with quantitative and count data, respectively. A multivariate logistic regression analysis was conducted to analyze the risk factors. *P* values < 0.05 were considered to be significant.

## Results

### Demographic Information

Table 3 shows basic demographic information of the HCWs who participated in this investigation. Ninety one of 138 HCWs (65.94%) had reported at least 1 incidence of ORAEs in the contaminated areas by March 26, 2020. Within 1073 entries into a contaminated area, 300 (27.96%) incidents of ORAEs were recorded. Table 4 shows the conditions of each person entering into contaminated areas. There are no significant differences among groups in terms of age, gender, occupation, qualification, weather, and temperature range (highest and lowest) conditions while entering contaminated areas, and the number of times worked in the contaminated areas. However, a longer duration of working in contaminated areas (4.15 ± 0.72) is significantly associated with ORAEs versus the duration (4.00 ± 0.80) of no ORAEs group, indicating that the length of working in contaminated areas may be a risk factor of developing ORAEs.

**Table 3.** Demographics of study participants

	No ORAEs (N = 47)	ORAEs (N = 91)	Total (N = 138)	t/χ^2^	*P*
Age	35 ± 5	36 ± 6	35 ± 6	t = 1.49	0.14
Male	8 (17.02%)	12 (13.19%)	20 (14.49%)	χ^2^ = 0.37	0.54
Female	39 (82.98%)	79 (86.81%)	118 (85.51%)		
Physician	13 (27.66%)	20 (21.98%)	33 (23.91%)	χ^2^ = 0.55	0.46
Nurse	34 (72.34%)	71 (78.02%)	105 (76.09%)		
Doctoral degree	9 (19.15%)	13 (14.29%)	22 (15.94%)	χ^2^ = 2.56	0.28
College degree	25 (53.19%)	61 (67.03%)	86 (62.32%)		
Master’s degree	13 (27.66%)	17 (18.68%)	30 (21.74%)		

**Table 4.** Conditions of HCWs entering contaminated area

Documented conditions	ORAEs (N = 300)	No ORAEs (N = 773)	Total (N = 1073)	t/χ^2^	*P*
Weather (sunny)	92 (30.67%)	257 (33.25%)	349 (32.52%)	χ^2^ = 0.77	0.69
Weather (cloudy)	42 (14.00%)	110 (14.23%)	152 (14.17%)		
Weather (rainy)	166 (55.33%)	406 (52.52%)	572 (53.31%)		
Temperature (high)	16.27 ± 3.79	16.06 ± 3.97	16.12 ± 3.92	T = −0.82	0.41
Temperature (low)	7.06 ± 3.59	6.77 ± 3.61	6.85 ± 3.60	T = −1.21	0.23
Duration (hours) in contaminated area	4.15 ± 0.72	4.00 ± 0.80	4.04 ± 0.78	T = 2.77	0.01*
Number of times in contaminated area	8 ± 7	8 ± 6	8 ± 7	T = −0.14	0.89

### Risk Factor Analysis

The incidence and rank of ORAEs documented in contaminated areas are shown in Table 5. Tenderness (205 times, 24.73% of all ORAEs), headache/dizziness (182 times, 21.95%), dyspnea (138 times, 16.65%), blurred vision (130 times, 15.68%), and nausea/vomiting (95 times, 11.46%) were the top 5 ORAEs documented.

**Table 5.** Frequencies, percent of total ORAEs, and incidences of various ORAEs in contaminated area

ORAEs	Frequencies	Percent Total (rank)	Incidence (rank)
Tenderness	205	24.73% (1)	19.10% (1)
Headache/dizziness	182	21.95% (2)	17.00% (2)
Dyspnea	138	16.65% (3)	12.90% (3)
Blurred vision	130	15.68% (4)	12.10% (4)
Nausea/vomiting	95	11.46% (5)	8.90% (5)
Palpitation	68	8.20% (6)	6.30% (6)
Emergencies (falls, epistaxis, stick/cut injuries)	11	1.33% (7)	1.00% (7)

**Figure 1.**
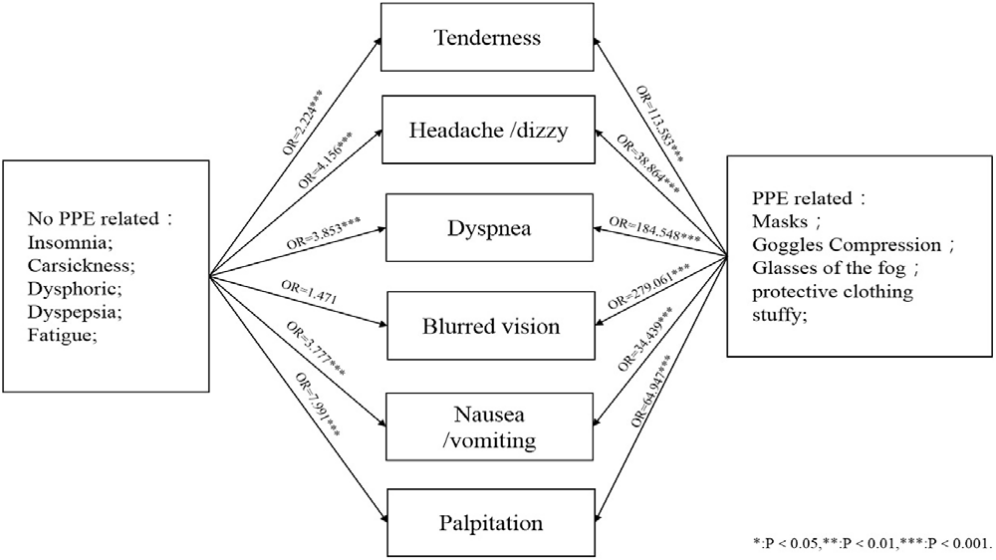
Relationship between ORAEs and related factors.

**Figure 2.**
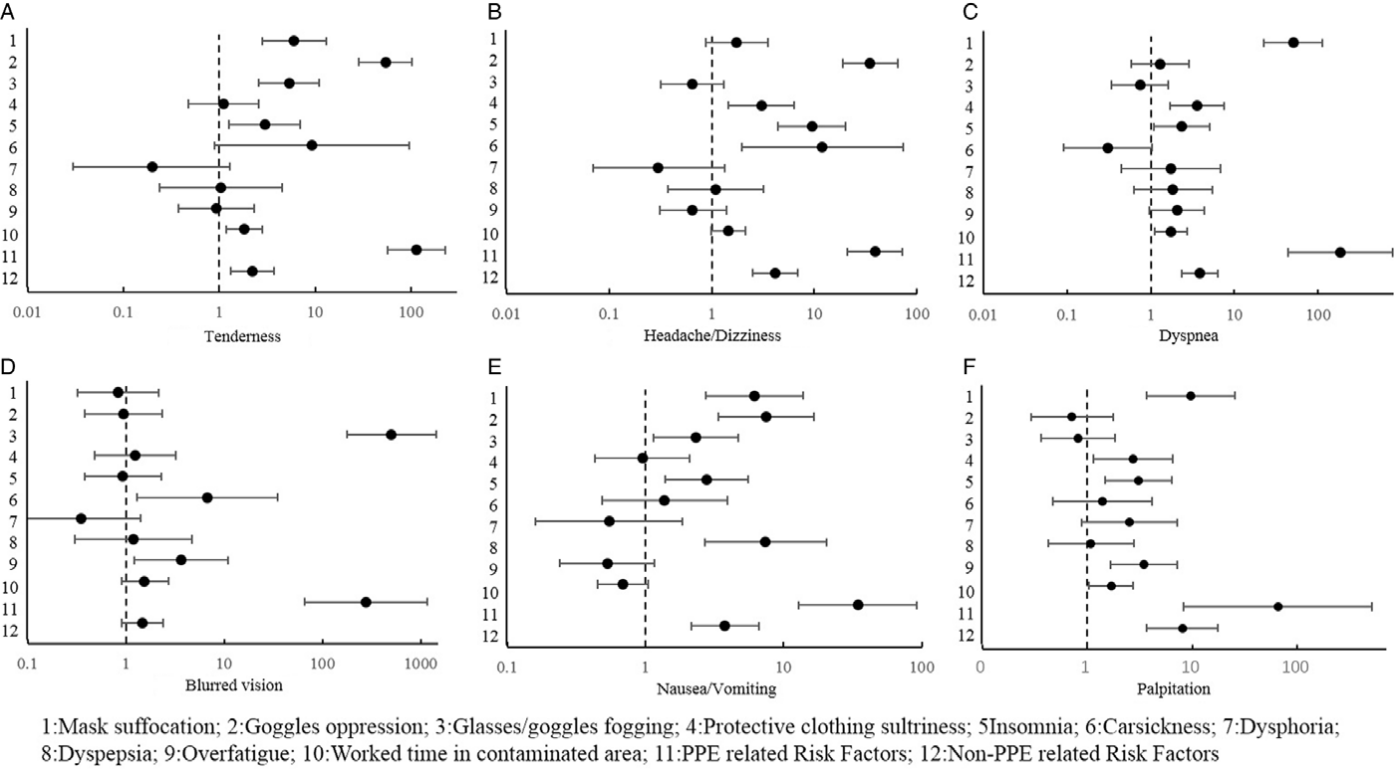
Logistic analysis of ORAEs and risk factors.

Relationships between ORAEs and presumed risk factors are shown in Figure 1. All ORAEs were significantly related to the use of PPE. Insomnia was associated with most ORAEs except blurred vision, although we are not certain whether it is the cause or the result of ORAEs, or both. Further detailed results are shown in Figure 2A to 2F.

Tenderness was mainly correlated with PPE-related factors, such as mask suffocation, goggles oppression, and glasses/goggles fogging. Work time in the contaminated area may be an independent risk factor for tenderness. For every 1 hour increased working in the contaminated area, the risk of tenderness would increase by 81.8%. Meanwhile, tenderness may be associated with insomnia, as shown in Figure 2A.

Headache and dizziness were correlated with goggles oppression, the sultriness of protective suit, insomnia, and car sickness, as shown in Figure 2B.

Dyspnea was correlated with mask suffocation, sultriness of protective suit, insomnia, and work time in the contaminated areas, as shown in Figure 2C.

Blurred vision was correlated with glasses/goggles fogging, overfatigue, and car sickness. In fact, overfatigue and car sickness may increase the risk of blurred vision in the contaminated areas, as shown in Figure 2D.

Mask suffocation, goggles oppression, and glasses/goggles fogging may also be correlated with nausea/vomiting in the contaminated areas, although the main reason was recent dyspepsia. Insomnia may also increase the risk of nausea/vomiting, as shown in Figure 2E.

Insomnia, overfatigue, mask suffocation, and protective suit sultriness may increase the risk of palpitation. For each hour increase in work time in a contaminated area, the likelihood of palpitation would increase by nearly 68.3%, as shown in Figure 2F.

The comparison of occupational exposure events and ORAEs pre- and post-interventions is shown in Tables 6 and 7. The incidence of occupational exposure events and the times of ORAEs in contaminated areas were both different pre- and post-interventions. The incidence of occupational exposure events before interventions was 0.4%, which was higher than post-intervention (0.05%); the difference was statistically significant (χ^2^=61.49, *P* < 0.001). The incidence density of ORAEs in contaminated areas before interventions was 73.15%, which was significantly higher (χ^2^ = 22.95, *P* < 0.001) than post-intervention (33.23%).

**Table 6.** Incidence of occupational exposure events before and after intervention

	Exposed	Unexposed	Incidence of Exposed
Pre-interventions	12	1848	0.60%
Post-interventions	13	17 807	0.1%
Total	25	19 655	0.1%
χ^2^	χ^2^ = 39.07		
*P*	*P* < 0.001		

**Table 7.** Prevalence of ORAEs in contaminated area before and after interventions

	ORAEs	No ORAEs	Incidence of ORAEs
Pre-interventions	79	108	73.15%
Post-interventions	221	665	33.23%
Total	300	773	38.81%
χ^2^	χ^2^ = 22.95		
*P*	*P* < 0.001		

## Discussion

The COVID-19 pandemic highlighted the necessity of health agencies worldwide to be well prepared to combat large-scale emerging infectious diseases. In the context of the outbreak of a novel severe respiratory infectious disease, especially at the early stage, reliable information of the pathogenic etiology and mechanism, mode of transmission, clinical presentation characteristics, and susceptible populations is lacking – and the availability of effective vaccines can be months to years away. To prevent cross-infection in hospitals, which may represent major spreading events of the infection and a significant impact to the occupational safety of HCWs, a standard and effective protocol of occupational protection is essential. Protective clothing, masks, and other PPE for HCWs entering wards to manage patients are already well accepted in the infectious disease units. In China, the dates when the peak of COVID-19 occurred were particularly unusual. It was during the traditional Spring Festival when manufacturing work was suspended almost all over China; thus, there was a marked shortage of production and supply of the PPEs. The vital role of medical care in the event of a huge number of patients, the fear of the unknown due to lack of sufficient information on the disease itself, and the pressure of public opinion place HCWs under huge psychological pressure. We experienced a stage when ORAEs, such as nausea/vomiting, headache/dizziness, tenderness, palpitation, dyspnea, and blurred vision, frequently occur in contaminated areas, and the incidence of occupational exposure, such as respiratory exposure, syncope, and needle stick injuries, was also relatively high, causing great physical and psychological burdens on HCWs.^5^

This study found that a long duration use of PPEs was the most important cause of ORAEs, which might be related to generally high-level protection while the effectiveness and necessity were not clearly defined. Although it is clearly not advisable to lower the protection level when the transmission mode of a novel severe respiratory infectious disease is unclear, optimizing the procedures of PPEs should be one of the key research areas to improve occupational safety for HCWs and enhance the quality of medical care, so that we can be more prepared for the emerging infectious diseases in the future. There are several key components of PPEs, and each may be improved to alleviate the risk of ORAEs. The protective clothing usually becomes sultry easily. Improving the air permeability of protective clothing material could be a practical way to reduce the burden of long-duration work while wearing these gowns. In practice, Tyvek medical protective clothing has excellent air permeability, with effective protectiveness, and has been highly approved for frontline HCWs.^6^ Mask suffocation is a major factor of ORAES. We think the following measures can be considered to reduce AEs: First, reducing the respiratory resistance by better manufacturing; second, decreasing the work duration and intensity by increasing the number of HCWs per shift would certainly improve the subjective feeling of mask suffocation; third, as irritating smells of masks can aggregate the perception of ORAEs, increasing the purity of the materials and improving the manufacturing process may help reduce some uncomfortable smells of masks. Many studies have reported that pressure injuries on skin associated with medical devices are caused by tightly worn goggles and masks. These injuries not only cause skin damage and pain, but also increase the risk of skin infection. We found that the use of protective dressing can reduce local friction, help absorb exudation, and promote repair while still ensuring that the airtightness of the mask and goggles are adjusted properly. If any pressure injuries are found, they should be properly treated. Antifogging agents applied to goggles are the most common way to prevent blurred vision. Although many goggles and protective face screens have antifogging features, moisture-resistant coatings are frequently destroyed by disinfectants. Povidone-iodine or detergents, such as handwashing solutions, may be reasonable substitutes when antifogging agents are in shortage. Anionic surfactants in detergents can reduce surface tension effectively to achieve moisture resistance; molecular iodine formed after povidone-iodine oxidation may also achieve moisture protection. Those alternative methods can generally achieve more than 2-hour antifogging effects, reducing the occurrence of ORAEs caused by blurred vision.^7^ Last, but not least, overuse and misuse of PPE should also be discouraged. Overuse of PPE by overlapping/double protective clothes, goggles, and masks results in a significant waste of already limited medical resources and increased disposal burden. Among non-PPE-related factors, HCWs were found to have a higher prevalence of insomnia, and it was also associated with most ORAEs in contaminated areas. Similar results have been reported in previous studies.^8–10^ When providing diagnoses and treatments to critically ill patients, due to frequent overtime work with higher intensity and occupational safety risk, HCWs would inevitably break sleep habits, which then lead to disruption of the biological clock. Long-term and persistent stressful events not only increase the risk of newly onset insomnia, but also enhance chronic insomnia. To mitigate the burden of insomnia for this group of people, the prevailing protocol is to deploy professional psychotherapists with active individual intervention, supplemented by the use of sedative hypnotic medicines. On the other hand, Car sickness is a risk factor that arises in a particular background. During the COVID-19 outbreak in Wuhan, HCWs were gathered in dormitories located more than 40 kilometers from the hospital and commuted with specially assigned shuttle buses so as to facilitate workforce management and to avoid cross-infection. As a result, multiple cases of ORAEs in contaminated areas associated with car sickness had been observed. Therefore, measures to reduce car sickness, such as reducing commute time by moving to closer lodging, training drivers to avoid operations that might induce car sickness, and distributing prophylactic pills to HCWs vulnerable to car sickness, may decrease the risk of ORAEs. Additionally, working duration in the contaminated “red zone” was also a crucial risk factor. The increased risk was associated not only with the clinical workload, but also the lengthy procedure to take off PPEs. There are potential risks of infection with several HCWS undressing PPE simultaneously within a limited space. Actually, there had been reports of infection events due to inappropriate PPE undressing every now and then.^11^^,^^12^ Therefore, HCWs had to undress PPEs sequentially following a strict protocol, which became a factor contributing to extended work time in contaminated areas. The problem became serious when numerous HCWs were required to enter the contaminated area at the same time as a large number of patients were admitted. Additional ameliorating measures to reduce risk of ORAEs for non-PPE related factors include setting up reasonable shift arrangement and increasing the number of HCWs per shift to alleviate fatigue and insomnia. In our experience, screening of AEs (eg, car sickness, insomnia) before entering contaminated areas, and appropriate intervention, when necessary, helped effectively reduce the negative impact of non-PPE-related factors. Needless to say, sufficient instructions and training on PPE on/off protocols are critical to ensure that HCWs are cultivating a good routine habit. One of the main reasons for occupational exposure at the early stage of the COVID-19 outbreak was insufficient protection for HCWs due to lack of PPE stocks and poorly implemented infection control measures. A study has confirmed that sufficient PPE could effectively decrease the occupational exposure of HCWs.^13^ Even if given sufficient supplies and assuming all being used adequately and reasonably, it’s still extremely important to ensure a thorough implementation of infection control measures, especially when many HCWs without much experience in dealing with infectious disease join the special workforce to combat the pandemic. For instance, by conveying the knowledge, improving the perception and behavior, strengthening the notion that “behavioral isolation” is superior to “physical isolation,” it is effective to maximize the contribution of PPEs in our experience.

One advantage of our bundled intervention model is the ability to make all aspects of the infection and AE risk controllable. Professional measures of infection control can not only decrease the infection rate,^14^ but also increase HCWs’ confidence to work against the viral infection. At the early stage of the pandemic, concerns and fears related to COVID-19 were prevalent among HCWs.^15^ So far, a majority of interventional research studies have assessed occupational exposure based on the SHEL model^16,17^ and the Reason model^18^; these methods analyzed occupational exposure caused by mismatchs between individual factors and the corresponding microenvironment from the perspective of system theory. The bundled interventions of this study were based on the findings of early investigations and then focused on taking actions against the most relevant issues. The bundled interventions had been effective, as indicated by the results of this study. Through these interventions, many HCWs became more compliant with the protection procedures; some incorrect perceptions, such as the notion that more and thicker PPEs offer better protection, were scientifically addressed. The results of our investigation and implementation in clinical practice indicate that this intervention model has high effectiveness and feasibility.

### Limitations

The limitations of this study are mainly reflected in 2 aspects. First, the self-control at different time points of clinical practice in this study is imperfect, as the HCWs are likely to get more experienced to deal with the daily routine at the later stage of this study. However, it is difficult to set a control to eliminate the influence of experience on our study results. Second, the statistical analyses of various types of ORAEs are limited by sample size. Thus, more intensive and in-depth studies are warranted in the future to support our results and conclusions.

## Conclusion

In summary, our study indicates that HCWs have suffered a high risk of occupational exposure and a high prevalence of ORAEs working in contaminated areas, related to various factors during the early stage of the COVID-19 epidemic. Timely, comprehensive, and multi-level interventions can significantly decrease the incidence of ORAEs and occupational exposures for HCWs working in contaminated areas.

## Supplementary material

For supplementary material accompanying this paper visit https://doi.org/10.1017/dmp.2021.237. click here to view supplementary material

## References

[1] Wan Mobin. Epidemic and epidemiological characteristics of SARS outbreak. Foreign Med Epidemiol Infect Dis Branch. 2003;3:129-132.

[2] Epidemiology Working Group for NCIP Epidemic Response, Chinese Center for Disease Control and Prevention. The epidemiological characteristics of an outbreak of 2019 novel coronavirus diseases (COVID-19) in China. Chin J Epidemiol. 2020;41(2):145-151.

[3] Special Expert Group for Control of the Epidemic of Novel Coronavirus Pneumonia of the Chinese Preventive Medicine Association. An update on the epidemiological characteristics of novel coronavirus pneumonia (COVID-19). Chin J Epidemiol. 2020;41(02):139-144. 10.3760/cma.j.issn.0254-6450.2020.02.002 32057211

[4] Guo ZD , Wang ZY , Zhang SF , et al. Aerosol and surface distribution of severe acute respiratory syndrome coronavirus 2 in hospital wards, Wuhan, China, 2020. Emerg Infect Dis. 2020;26(7). 10.3201/eid2607.200885 PMC7323510 32275497

[5] Tang H , Lu X , Cai S , et al. Investigation and analysis on mental health status of frontline nurses in Wuhan during COVID-19 epidemic. Int Infect Dis. 2020;9(02):296-297.

[6] Lu L , Zhang H . Review of the advancement in protective clothing comfort. J Beijing Instit Cloth Technol. 2014, 034(003):38-45.

[7] Li L , Cao X . Application and effect observation of different protective eyepiece anti-fog methods in COVID-19 care isolation Ward. J Nurses Train. 2020;35(9):817-819.

[8] Nicolle L . SARS safety and science. Can J Anaesth. 2003;50(10):983. 14656774 10.1007/BF03018360 PMC7090529

[9] Cai J , Qiao A , Bai Y , et al. Status quo and influencing factors of the sleep status of medical staff fighting against COVID-2019 in Huoshenshan Hospital. Hosp Adm J Chin PLA. 2020;27(3).

[10] China Sleep Research Association. Chinese guidelines for diagnosis and treatment of insomnia. Chin Med J. 2017;97(24):1844-1856.

[11] China News. Spanish Ebola nurse is feared to have been infected by misuse of protective equipment. 2021/6/3. http://www.chinanews.com/gj/2014/10-09/6659093.shtml

[12] World Health Organization. WHO was informed of the first confirmed autochthonous case of Ebola virus disease (EVD) in Spain. This case represents the first human-to-human transmission of EVD outside Africa. Published October 9, 2014. Accessed June 3, 2021. https://www.who.int/emergencies/disease-outbreak-news/item/09-october-2014-ebola-en

[13] Chen M , Wei X , Wang Z . Protecting healthcare workers from SARS-CoV-2 and other infections. *Epidemiol Infect*. 2020;e217:1-4. 10.1017/S0950268820002198 PMC7525083 32951627

[14] Correa-Martínez CL , Schwierzeck V , Mellmann A , et al. Healthcare-associated SARS-CoV-2 transmission – experiences from a German university hospital. Microorganisms. 2020;8(9):1378. 10.3390/microorganisms8091378 PMC7563154 32911751

[15] Shreffler J , Petrey J , Huecker M . The impact of COVID-19 on healthcare worker wellness: a scoping review. West J Emerg Med. 2020, 21(5):1059-1066. 32970555 10.5811/westjem.2020.7.48684 PMC7514392

[16] Guo X , Lu C , Wang Y , et al. Application of SHEL model in protection of occupational exposure during epidemic of COVID-19 among nurses. Chin Nurs Res. 2020;34(06):943-944.

[17] Molloy GJ , O’Boyle CA . The SHEL model: a useful tool for analyzing and teaching the contribution of human factors to medical error. Acad Med. 2005;80(2):152-155. 15671319 10.1097/00001888-200502000-00009

[18] Reason J . Human error: models and management. West J Med. 2000;172(6):393-396. 10854390 10.1136/ewjm.172.6.393 PMC1070929

